# Early moderate exercise benefits myocardial infarction healing via improvement of inflammation and ventricular remodelling in rats

**DOI:** 10.1111/jcmm.14710

**Published:** 2019-10-15

**Authors:** Zhaofu Liao, Dan Li, Yilin Chen, Yunjian Li, Ruijin Huang, Kuikui Zhu, Hongyi Chen, Ziqiang Yuan, Xin Zheng, Hui Zhao, Qin Pu, Xufeng Qi, Dongqing Cai

**Affiliations:** ^1^ Key Laboratory of Regenerative Medicine Ministry of Education Jinan University Guangzhou China; ^2^ Joint Laboratory for Regenerative Medicine Chinese University of Hong Kong‐Jinan University Guangzhou China; ^3^ International Base of Collaboration for Science and Technology (JNU) The Ministry of Science and Technology & Guangdong Province Guangzhou China; ^4^ Department of Developmental & Regenerative Biology Jinan University Guangzhou Guangzhou China; ^5^ Department of Neuroanatomy Institute of Anatomy University of Bonn Bonn Germany; ^6^ Department of Anatomy and Molecular Embryology Institute of Anatomy and Cell Biology University of Freiburg Freiburg Germany; ^7^ Department of Medical Oncology Cancer Institute of New Jersey Robert Wood Johnson of Medical School New Brunswick NJ USA; ^8^ Stem cell and Regeneration TRP School of Biomedical Sciences Chinese University of Hong Kong Hong Kong

**Keywords:** cardiac telocytes, early post‐MI moderate exercise, inflammation, myocardial infarction, remodelling

## Abstract

Thus far, the cellular and molecular mechanisms related to early (especially within 24 hours after acute myocardial infarct (MI)) exercise‐mediated beneficial effects on MI have not yet been thoroughly established. In the present study, we demonstrated that acute MI rats that underwent early moderate exercise training beginning one day after MI showed no increase in mortality and displayed significant improvements in MI healing and ventricular remodelling, including an improvement in cardiac function, a decrease in infarct size, cardiomyocyte apoptosis, cardiac fibrosis and cardiomyocyte hypertrophy, and an increase in myocardial angiogenesis, left ventricular wall thickness and the number of cardiac telocytes in the border zone. Integrated miRNA‐mRNA profiling analysis performed by the ingenuity pathway analysis system revealed that the inhibition of the TGFB1 regulatory network, activation of leucocytes and migration of leucocytes into the infarct zone comprise the molecular mechanism underlying early moderate exercise‐mediated improvements in cardiac fibrosis and the pathological inflammatory response. The findings of the present study demonstrate that early moderate exercise training beginning one day after MI is safe and leads to significantly enhanced MI healing and ventricular remodelling. Understanding the mechanism behind the positive effects of this early training protocol will help us to further tailor suitable cardiac rehabilitation programmes for humans.

## 1 INTRODUCTION

Myocardial infarction (MI) is a major cause of human morbidity and mortality worldwide.^1^ Patients who survive MI frequently develop systolic heart failure because of the infarct‐induced loss of functional myocardium per se and the remodelling of the left ventricle (LV), which involves cardiomyocyte necrosis, cardiomyocyte hypertrophy, LV wall thinning, infarct expansion and collagen accumulation. Although several therapeutic approaches have been proven to ameliorate post‐infarction cardiac remodelling, the prognosis remains poor, and LV dysfunction often progresses to heart failure.^2^ For many years, avoiding physical activity after MI was recommended; however, the current view proposes that moderate exercise training should be a part of cardiac rehabilitation programmes. There is increasing evidence that exercise training, including exercise training before and after infarction, provides promising effects on the repair of the infarcted heart, regardless of the decrease in infarct size and cardiac fibrosis, the attenuation of apoptosis in the myocardium, improvements in ventricular remodelling and inflammation.^3^, ^4^, ^5^, ^6^, ^7^, ^8^, ^9^, ^10^

However, various studies in animals and humans have also shown conflicting results concerning the effects of exercise training, including neutral^11^, ^12^, ^13^, ^14^ and adverse^15^, ^16^, ^17^ effects, on LV remodelling after MI. The variation of the effects might be due to differences in exercise intensity after MI. High‐intensity exercise can overload the infarcted heart, whereas low‐intensity exercise exerts little if any physiological impact. The timing at which exercise training starts after MI might also be a critical factor in its effect on regeneration after MI. Studies in rats have indicated that exercise that is started late (more than three weeks after MI) does not aggravate^13^, ^18^ or blunts^4^, ^6^, ^19^ LV dilation and hypertrophy, whereas exercise started less than a week after MI results in beneficial,^20^ no^11^, ^21^ or detrimental^15^, ^16^ effects on LV remodelling. In a more recent human randomized controlled trial study, it was documented that early exercise‐based rehabilitation in which patients underwent a supervised early mobilization exercise programme twice a day beginning 12 hours after acute MI (the inpatient phase) improved health‐related quality of life and functional capacity in patients with low cardiovascular risk who experienced acute MI.^22^ In rats that experienced a large induced MI, early voluntary exercise training (started within 24 hours after MI) had no impact on survival or LV remodelling but attenuated global LV dysfunction^23^ and restored cardiomyocyte contraction via improving the myofilament Ca^2+^ response and diastolic Ca^2+^ handling.^24^ In this study, a voluntary exercise programme in which rats ran approximately 5 km a day on average was utilized. It is not yet clear whether and how early exercise of moderate intensity (600 m/d)^25^ beginning one day after MI, in contrast to voluntary exercise (approximately 5 km/d on average), benefits the repair of the infracted heart.

In addition, it has been documented that the number of cardiac telocytes increases significantly in the heart following a 4‐week ramp swimming exercise programme in mice.^26^ These findings suggest that cardiac interstitial cells, such as cardiac telocytes, might be involved in exercise‐mediated beneficial effects in the myocardium and might promote the healing and regeneration of the damaged myocardium.

The present study was therefore designed to evaluate the underlying mechanism of the beneficial effects mediated by moderate exercise conducted one day after MI and mainly focused on the effects of exercise on the inflammatory response, ventricular remodelling and cardiac telocyte density in MI.

## 2 MATERIALS AND METHODS

### 2.1 Animals

Three‐month‐old female Sprague Dawley rats (250‐300 g) were utilized in the present study. Animal care, surgery and handling procedures were performed according to regulations established by the Ministry of Science and Technology of the People's Republic of China ([2006] 398) and were approved by the Jinan University Animal Care Committee.

### 2.2 Myocardial infarction induction

Myocardial infarct was generated through left anterior descending coronary artery (LAD) ligation in three‐month‐old female Sprague Dawley rats, as previously described.^27^, ^28^ For details, see Appendix S1.

### 2.3 Treadmill exercise

**Figure 1:**
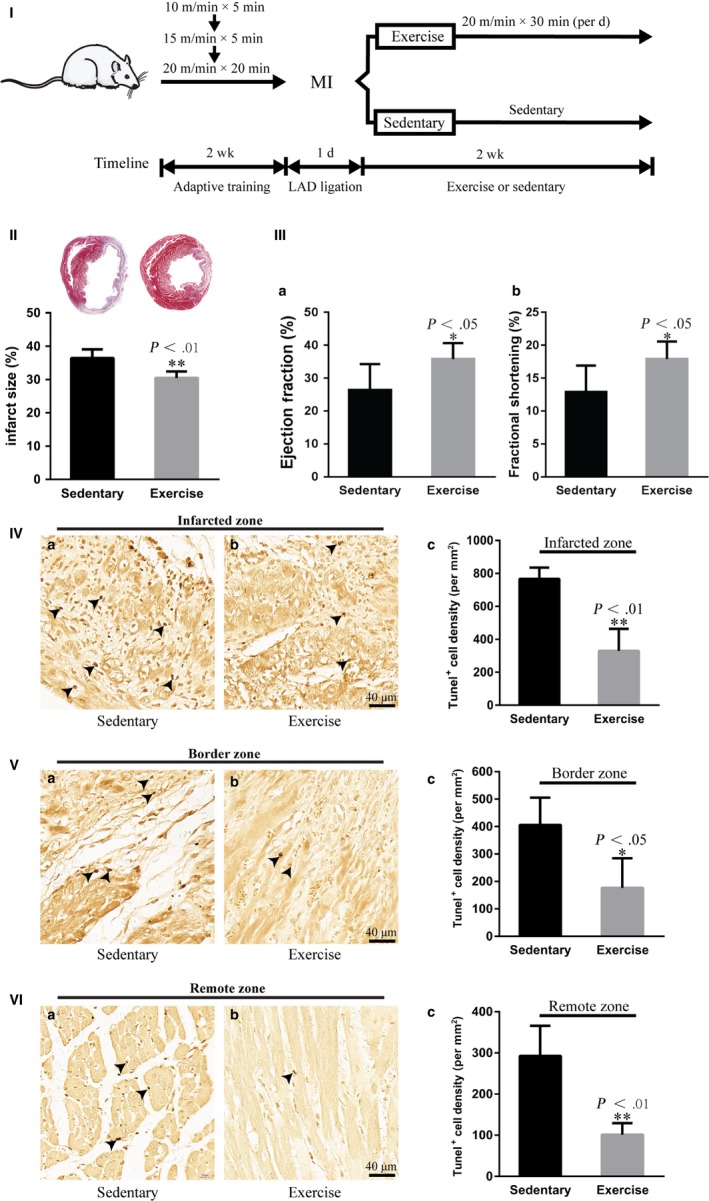
Early moderate exercise reduces infarct size and cardiomyocyte apoptosis and improves cardiac function in myocardial infarct (MI). (I) Schematics of the training programme. (II) Masson's trichrome staining showed that early moderate exercise reduced infarct size. n = 6‐8. (III) Echocardiography revealed that early moderate exercise improved the ejection fraction (a) and fractional shortening (b). n = 5‐8. (IV‐VI) TUNEL staining demonstrated that early moderate exercise attenuated cardiomyocyte apoptosis. (a) Sedentary heart. (b) Moderate exercise heart. (c) Semiquantification of (a) and (b). n = 4‐5. For early moderate exercise, the animals were trained for two weeks beginning one day after MI

All animals were habituated to a motorized treadmill by running following a gradual acceleration protocol (0° grade; 10 m/min for 5 minutes, 15 m/min for 5 minutes and 20 m/min for 20 min) each day for 2 weeks. Following 2 days of rest, the rats were randomly divided into two sets: set‐1, a sedentary control group (n = 20) and a moderate exercise group (n = 23); set‐2, a sedentary control group (n = 7) and a moderate exercise group (n = 10). Both sets underwent LAD ligation to induce MI as described above. The rats in the moderate exercise group ran on the treadmill beginning one day after MI for 2 weeks using the moderate exercise protocol, which was set at 20 m/min for 30 min/d (hereafter called the exercise group).^25^ The rats in the sedentary control group were allowed to be sedentary in their cages for 2 weeks after MI (Figure 1I). The set‐1 animals were used for all observations except the cardiac function analysis, whereas the set‐2 animals were used to analyse of cardiac function. Both sets of animals were included to analyse mortality rate.

### 2.4 Echocardiography

Cardiac function was evaluated by echocardiography as described in Appendix S1.

### 2.5 Histological staining

Masson's trichrome staining, the TUNEL assay and immunohistochemistry were performed as described in Appendix S1.

### 2.6 mRNA and miRNA sequencing

After 2 weeks training or sedentary behaviour, three infarcted hearts in each group were used for sequencing analysis. Total RNA was extracted from the infarct zone using QIAGEN RNeasy Kits (QIAGEN, 217004). RNA integrity was evaluated using an Agilent 2100 Bioanalyzer.

For mRNA sequencing, the libraries were constructed using a TruSeq Stranded mRNA Library Prep Kit (Illumina, RS122‐2101) and sequenced on an Illumina sequencing platform (HiSeqTM4000, Illumina). Paired‐end reads (150 bp) were generated and mapped to the rat genome (Rnor_5.0) using TopHat, and gene expression was estimated as fragments per kb per million reads. Differentially expressed genes were identified using DESeq software.

For miRNA sequencing, libraries were constructed using a TruSeq Small RNA Sample Prep Kit (Illumina, RS200‐0024) and sequenced on an Illumina sequencing platform (HiSeqTM4000, Illumina). After filtration, clean reads were compared with miRNA databases (miRbase 20.0) to annotate the mature small RNA sequences by Bowtie. miRNA expression was estimated as reads per million.

mRNAs and miRNAs with differential expression equal to or greater than a two‐fold change were considered to be differentially expressed and were selected for ingenuity pathway analysis (IPA). For details, see Appendix S1.

### 2.7 mRNA‐miRNA integrative IPA

The selected mRNAs and miRNAs with a log_2_ expression ratio greater than 1 or less than −1 were used for further mRNA‐miRNA integrative analysis to identify regulatory networks and disease function analysis using IPA (http://www.ingenuity.com). The analysis results were used for an additional core analysis in the IPA system. In the present study, only the analysed data that were used to predict “decrease” or “increase”, which was indicated by a *P* value <.001 and a *z*‐score larger than 2 or less than −2, were selected as positive predictors. For details, see Appendix S1.

### 2.8 Real‐time quantitative PCR

Gene and miRNA expression levels were analysed using SYBR green‐based quantitative PCR (qPCR). Relative expression was determined using the 2^−ΔΔCt^ comparative threshold method. For details, see Appendix S1. The primer list is shown in Appendix S2.

### 2.9 Statistics

All measured data are presented as the means ± standard errors. Two‐tailed Student's *t* test was used to calculate the statistical significance between two groups. *P* values <.05 were considered significant.

## 3 RESULTS

### 3.1 Early moderate exercise does not affect mortality

After LAD ligation to induce MI, 43 LAD‐ligated rats were randomly divided into the early moderate exercise group (n = 23) and the sedentary control group (n = 20). The mortality rates were 21.74% (5/23) and 20.00% (4/20) in the early moderate exercise group and the sedentary control group, respectively. A set of LAD‐ligated rats (n = 17), 10 rats from the early moderate exercise group and 7 rats from the sedentary group, were used to compare cardiac function and further confirm the difference in mortality rate between animals that underwent exercise and those that did not. The mortality rate of the early moderate exercise group (2/10; 20%) was even lower than that of the sedentary control group (2/7; 28.57%). The total mortality rates of the two sets were 21.21% (7/33) and 22.22% (6/27) in the early moderate exercise group and the sedentary control group, respectively.

### 3.2 Early moderate exercise reduces infarct size and apoptosis of cardiomyocytes and improves cardiac function in acute MI

The infarct size in the exercise group was significantly smaller than that in the sedentary group (Figure 1II; *P* < .01). To determine whether the decreased infarct size was related to decreased apoptosis, TUNEL staining was used to compare apoptosis between the exercise and sedentary groups. Our TUNEL staining results revealed that the densities of apoptotic cardiomyocytes in the infarct zone, border zones and remote noninfarcted zone in the exercise group were significantly lower than those in the sedentary group (Figure 1IV‐VI; *P* < .01; *P* < .05; *P* < .01). Importantly, it was found that cardiac function, including ejection fraction (EF) and fractional shortening (FS) in the early moderate exercise group, was significantly improved compared with that in the sedentary group (Figure 1III; *P* < .05).

### 3.3 Early moderate exercise improves angiogenesis, fibrosis and ventricular remodelling after MI

**Figure 2:**
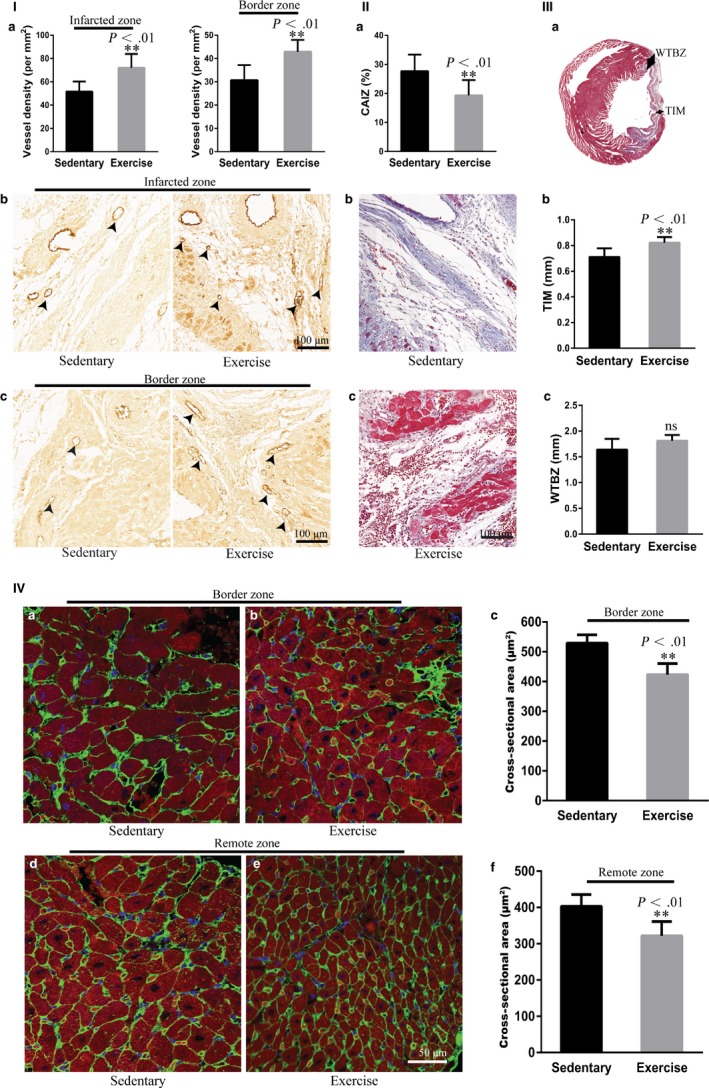
Early moderate exercise improves angiogenesis, fibrosis and ventricular remodelling in myocardial infarct (MI). (I) Immunohistochemical staining for vWF showed that blood vessel density in the infarct zone and border zone in early moderate exercise hearts was significantly higher than those in sedentary hearts. (a) Semiquantification of (b) and (c). (b) Infarct zone. (c) Border zone. n = 6‐8. (II) Masson's trichrome staining documented that the collagen area of the infarct zone in early moderate exercise hearts was significantly smaller than that in sedentary hearts. (a) Semiquantification of (b) and (c). (b) Sedentary heart. (c) Early moderate exercise heart. n = 9 per group. (III) Masson's trichrome staining revealed that the TIM in early moderate exercise hearts was significantly larger than that in sedentary hearts, whereas the difference in the WTBZ between early moderate exercise hearts and sedentary hearts was not significant. (a) Schematics of TIM and WTBZ. (b) Semiquantification of TIM. (c) Semiquantification of WTBZ. n = 6‐9 per group. (IV) WAG immunofluorescence staining demonstrated that the cardiomyocyte cross‐sectional area of both the border zone and the remote zone in early moderate exercise hearts was significantly smaller than those in sedentary hearts. (a) Border zone of the sedentary heart. (b) Border zone of the early moderate exercise heart. (c) Semiquantification of (a) and (b). (d) Remote zone of the sedentary heart. (e) Remote zone of the early moderate exercise heart. (f) Semiquantification of (d) and (e). n = 5 per group. The animals were trained on early moderate exercise for two weeks beginning one day after MI

In the present study, the effect of early moderate exercise on post‐MI regeneration was evaluated in terms of angiogenesis, fibrosis and the remodelling of the infracted heart. The vWF immunohistochemistry results showed that the blood vessel density in the infarct and border zone in the exercise group was significantly higher than that in the sedentary group (Figure 2I; *P* < .01; *P* < .01). The antifibrotic effect was evaluated by comparing the collagen area of the infarct zone (CAIZ). The CAIZ in the exercise group was significantly smaller than that in the sedentary group (Figure 2II; *P* < .01). The effect of early moderate exercise on post‐infarct remodelling was determined by measuring the thickness of the infarcted myocardium of the left ventricle (TIM) and the wall thickness of the border zone of the left ventricle (WTBZ). The TIM in the exercise group was significantly larger than that in the sedentary group (Figure 2IIIb; *P* < .01), whereas the difference in the WTBZ between both groups was not significant (Figure 2IIIc; *P* > .05). It is well established that following acute MI, cardiomyocyte volume is enlarged as an attempt to compensate for the loss of myocardial mass. Therefore, we next examined the cardiomyocyte cross‐sectional area (CSA), which was identified by wheat‐germ agglutinin (WGA) immunofluorescence staining in both the remote zone and the border zone after MI. The CSAs of both the border zone and the remote zone in the exercise group were significantly smaller than those in the sedentary group (Figure 2IV; *P* < .01; *P* < .01).

### 3.4 Early moderate exercise increases the number of cardiac telocytes in the border zone but not in the infarct zone

**Figure 3:**
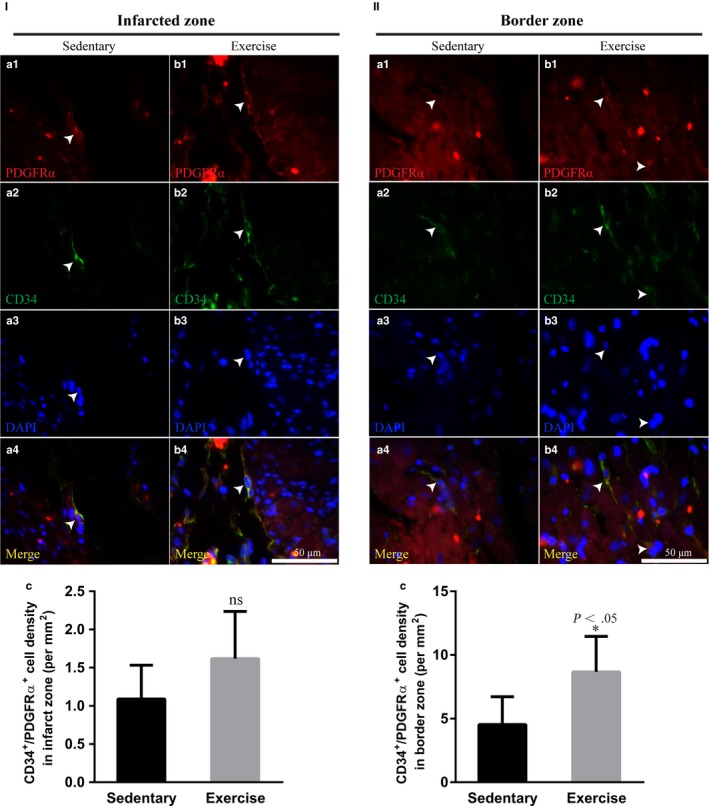
Early moderate exercise increases the number of cardiac telocytes in the border zone but not in the infarct zone. (I) Immunofluorescence staining for PDGFRα^+^/CD34^+^ revealed that the density of CTs in the early moderate exercise group was slightly increased compared with that in the sedentary group; however, the difference was not statistically significant. (II) Immunofluorescence staining for PDGFRα^+^/CD34^+^ revealed that, in the border zone, the density of CTs in the early moderate exercise group was significantly increased compared with that in the sedentary group. (a1‐4) Sedentary heart. (b1‐4) Early moderate exercise heart. (c) Semiquantification of (I) and (II). n = 5 per group. The animals were trained on early moderate exercise for two weeks beginning one day after MI

As interstitial cells, cardiac telocytes (CTs) have been recently identified as a distinct interstitial cell type that exists in heart and other tissues and organs.^29^, ^30^, ^31^, ^32^, ^33^ We previously reported that CTs are distributed longitudinally and within the cross‐network of the myocardium, which experiences serious cell death and is significantly decreased in the infarct zone after acute MI in rats.^34^, ^35^ In addition, the number of CTs in the heart has been reported to be increased significantly following a 4‐week ramp swimming exercise programme in mice.^26^ These data suggest that CTs might be involved in exercise‐mediated beneficial effects on MI. To reveal the CT networks in the MI heart, cells positive for PDGFRα and CD34, two makers generally used to identify CTs,^26^, ^31^ were identified by using immunofluorescence staining. In the infarct zone, the density of CTs was slightly increased in the exercise group compared with the sedentary group (Figure 3I; *P* > .05). However, in the border zone, the density of CTs was significantly increased in the exercise group compared with the sedentary group (Figure 3II; *P* < .05).

### 3.5 Early moderate exercise inhibits the inflammatory response in the infracted myocardium

**Figure 4:**
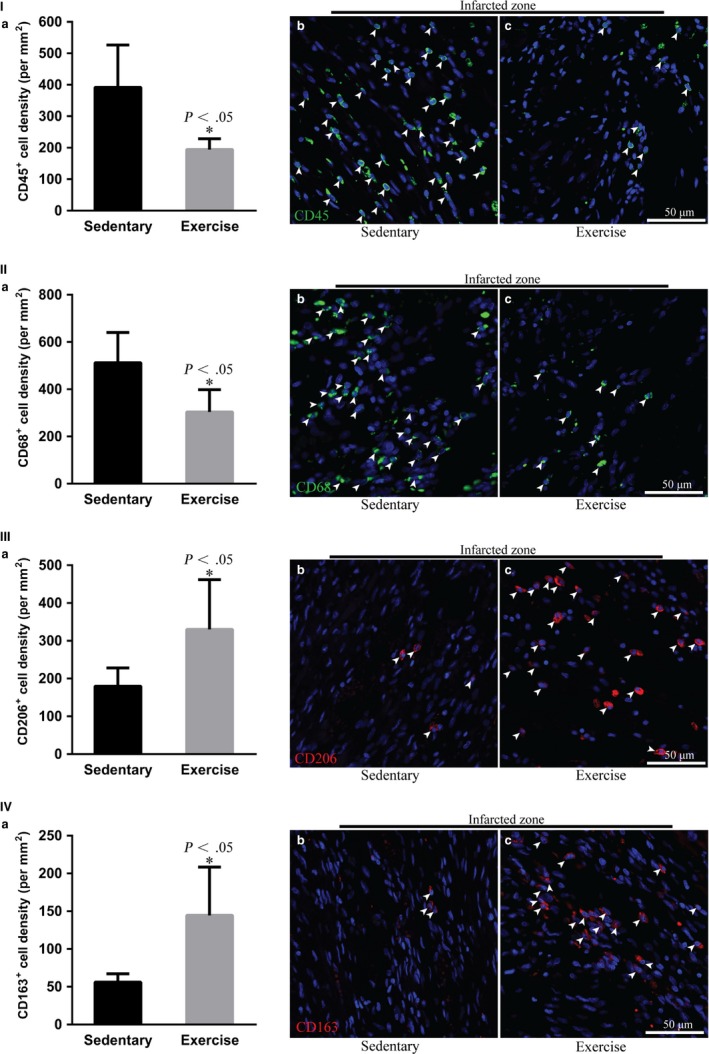
Early moderate exercise inhibits the inflammatory response in infarcted myocardium. (I) Immunofluorescence staining for CD45^+^ leucocytes revealed that the density of CD45^+^ leucocyte infiltration in the infarct zone was significantly decreased in early moderate exercise hearts compared with sedentary hearts. (II) Immunofluorescence staining for CD68^+^ macrophages (M1 macrophages) demonstrated that the density of M1 macrophages in the infarct zone was significantly decreased in early moderate exercise hearts compared with sedentary hearts. (III) Immunofluorescence staining for CD206^+^ macrophages (M2) showed that the density of CD206^+^ macrophages in the infarct zone was increased significantly in early moderate exercise hearts compared with sedentary hearts. (IV) Immunofluorescence staining for CD163^+^ macrophages (M2) showed that the density of CD163^+^ macrophages in the infarct zone was increased significantly in early moderate exercise hearts compared with sedentary hearts. (a) Semiquantification of (b) and (c). (b) Sedentary heart. (c) Early moderate exercise heart. n = 5 per group. The animals were trained on early moderate exercise for two weeks beginning one day after MI

To determine whether early moderate exercise has beneficial effects on the inflammatory response in MI, we assessed the characteristics of inflammatory cells, including pro‐inflammatory cells, namely CD45^+^ leucocytes and CD68^+^ macrophages (M1 macrophages), and anti‐inflammatory cells, namely CD206^+^ macrophages and CD163^+^ macrophages (M2 macrophages), in the infarct zone. The density of CD45^+^ leucocyte and CD68^+^ macrophages infiltration in the infarct zone was significantly decreased in the exercise group compared with the sedentary group (Figure 4I,II; *P* < .05). In contrast, the density of CD206^+^ macrophages and CD163^+^ macrophages infiltration in the infarct zone was significantly increased in the early moderate exercise group compared with the sedentary group (Figure 4III,IV; *P* < .05).

### 3.6 miRNA‐mRNA integrative IPA reveals that the inhibition of the TGFB1 regulatory network is a major underlying mechanism of the early moderate exercise‐mediated improvement of cardiac fibrosis and ventricular architecture remodelling in MI

To reveal the underlying molecular mechanism of the significant promotion of MI healing and ventricular remodelling that was achieved by initiating early moderate exercise, we first applied IPA to analyse 964 genes that were differentially expressed in the infract zone between the early moderate exercise MI hearts and the sedentary MI hearts and that were identified by transcriptome sequencing. IPA showed no predicted activation or inhibition effects regarding the canonical pathway, upstream signalling, molecular and physiological function, and disease toxicity. However, IPA of canonical pathway analysis revealed that the Th2 pathway and leucocyte extravasation signalling in the infarct zone of early moderate exercise MI hearts tended to be activated and inhibited, respectively, compared with those in the sedentary MI heart (*z*‐score < 2). The related genes are listed in Appendix S3. Recent progress has demonstrated that miRNAs might play a critical role in exercise‐mediated beneficial regulation in MI.^36^ Therefore, we also applied IPA to analyse the differential expression of 87 miRNAs in the infarct zone between early moderate exercise MI hearts and sedentary MI hearts. IPA showed that, at the miRNA level, no predicted activation or inhibition effects regarding the canonical pathway, upstream signalling, or molecular and physiological function were identified (*z*‐score < 2). Conversely, IPA of disease toxicity function identified some miRNAs that are related to cardiotoxicity (*z*‐score < 2), such as miR‐150‐5p and miR‐133a‐3p, which are involved in the regulation of cardiac fibrosis and cardiac hypertrophy, as shown in Appendix S4.

**Figure 5:**
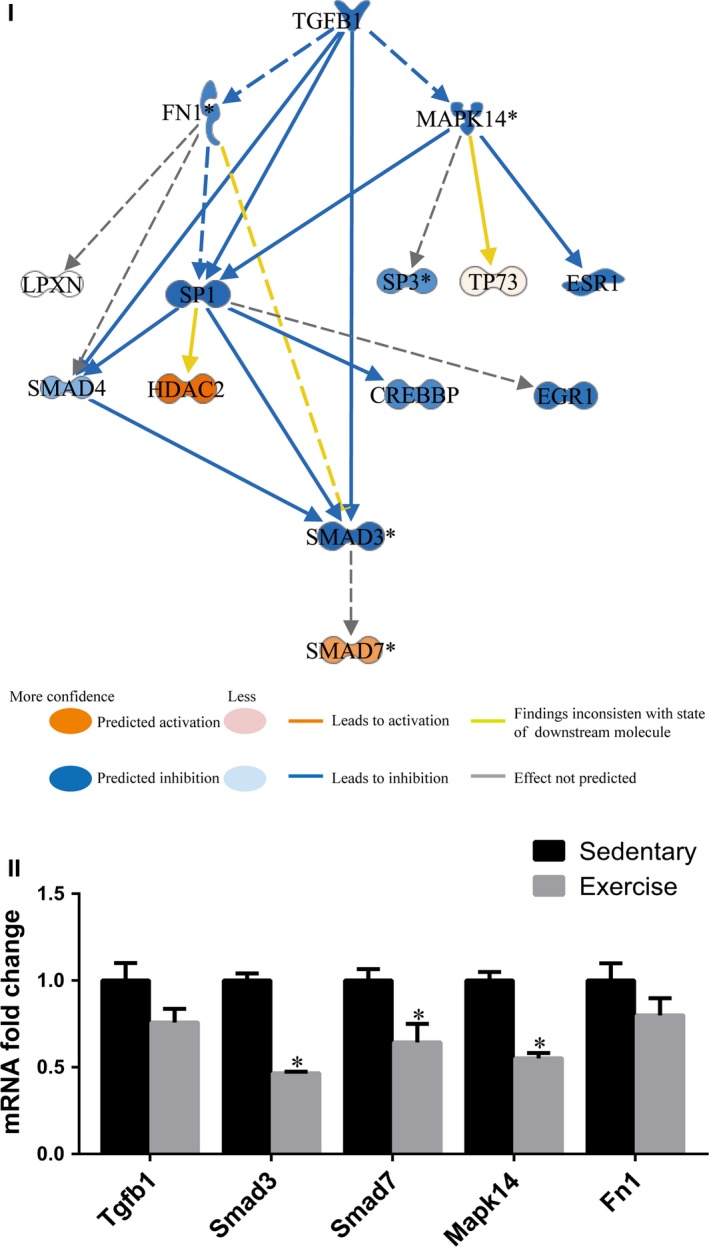
Inhibition of the TGFB1 regulatory network in the infarct zone of the early moderate exercise‐trained heart. (I) The upstream analysis of the integrated ingenuity pathway analysis between miRNAs and mRNAs in the infarct zone predicted that compared with the sedentary MI heart, the primary upstream molecule was *TGFB1*, which was inhibited in the infarct zone of the early moderate exercise MI heart (*P*‐value = 1.78E‐08; *z*‐score = −2.266). The inhibition of 10 genes (*TGFB1*, *FN1*, *MAPK14*, *SP1*, *SP3*, *ESR1*, *SMAD4*, *EGR1*, *CREBBP* and *SMAD3*) and the activation of 3 genes (*HDAC2*, *TP73* and *SMAD7*) played a role in the inhibition of the *TGFB1* regulatory network. The network displays the regulatory relationship of these 13 genes in which *SP1*, *SMAD4* and *SMAD3*, the direct downstream genes that are regulated by *TGFB1*, were inhibited, whereas *SMAD7*, the inhibitor of the *TGFB1* pathway, was activated. In addition, the indirect downstream genes, *FN1*, *MAPK14*, *SP3*, *ESR1*, *EGR1* and *CREBBP*, were predicted to be inhibited, whereas *TP73* was activated. (II) The qPCR quantifications of the expression levels of selected genes included in the *TGFB1* network. The animals were trained on early moderate exercise for two weeks beginning one day after MI. n = 3 per group. *P < .05 vs the sedentary group

It is well established that the functional role of miRNAs is mainly to modulate gene expression through both mRNA degradation and translational repression mechanisms^37^, ^38^; therefore, the integrated analysis of differential expression profiles of miRNAs‐mRNAs between early moderate exercise hearts and sedentary hearts in the infarct zone might allow us to uncover the molecular mechanism underlying the early exercise‐mediated beneficial effects on facilitating healing and regeneration after MI, which were demonstrated in the present study. For this purpose, we used the 964 genes and 87 miRNAs that were differentially expressed between early moderate exercise hearts and sedentary hearts and that were identified by whole transcriptome sequencing and small RNA sequencing to conduct miRNA‐mRNA integrative pair analysis using the IPA system. According to the literature, the IPA system is thus far the most authoritative tool for achieving this type of analysis. The upstream integrated analysis of the integrated IPA between miRNAs and mRNAs predicted that, compared with that in sedentary hearts, the regulatory role of *TGFB1* in the infarct zone of exercise hearts was inhibited (*P*‐value = 1.78E‐08; *z*‐score = −2.266), and this was attributed to the inhibition of 10 genes (*TGFB1*, *FN1*, *MAPK14*, *SP1*, *SP3*, *ESR1*, *SMAD4*, *EGR1*, *CREBBP* and *SMAD3*) and the activation of 3 genes (*HDAC2*, *TP73* and *SMAD7*) (Figure 5I). Indeed, qPCR analysis of the selected representative genes confirmed that the expression levels of *SMAD3* and *MAPK14* were significantly down‐regulated in the infarct zone in exercise hearts compared with sedentary hearts (*P* < .05). In contrast, the expression levels of *TGFB1* and *FN1* tended to be down‐regulated (Figure 5II). IPA also revealed that 74 genes (63 down‐regulated genes and 11 up‐regulated genes) are included in the regulatory network of TGFB1 inhibition, as shown in Appendix S5.

### 3.7 miRNA‐mRNA integrative IPA reveals that the inhibition of leucocyte activation and migration is the other major underlying mechanism by which early moderate exercise improves the inflammatory response in MI

**Figure 6:**
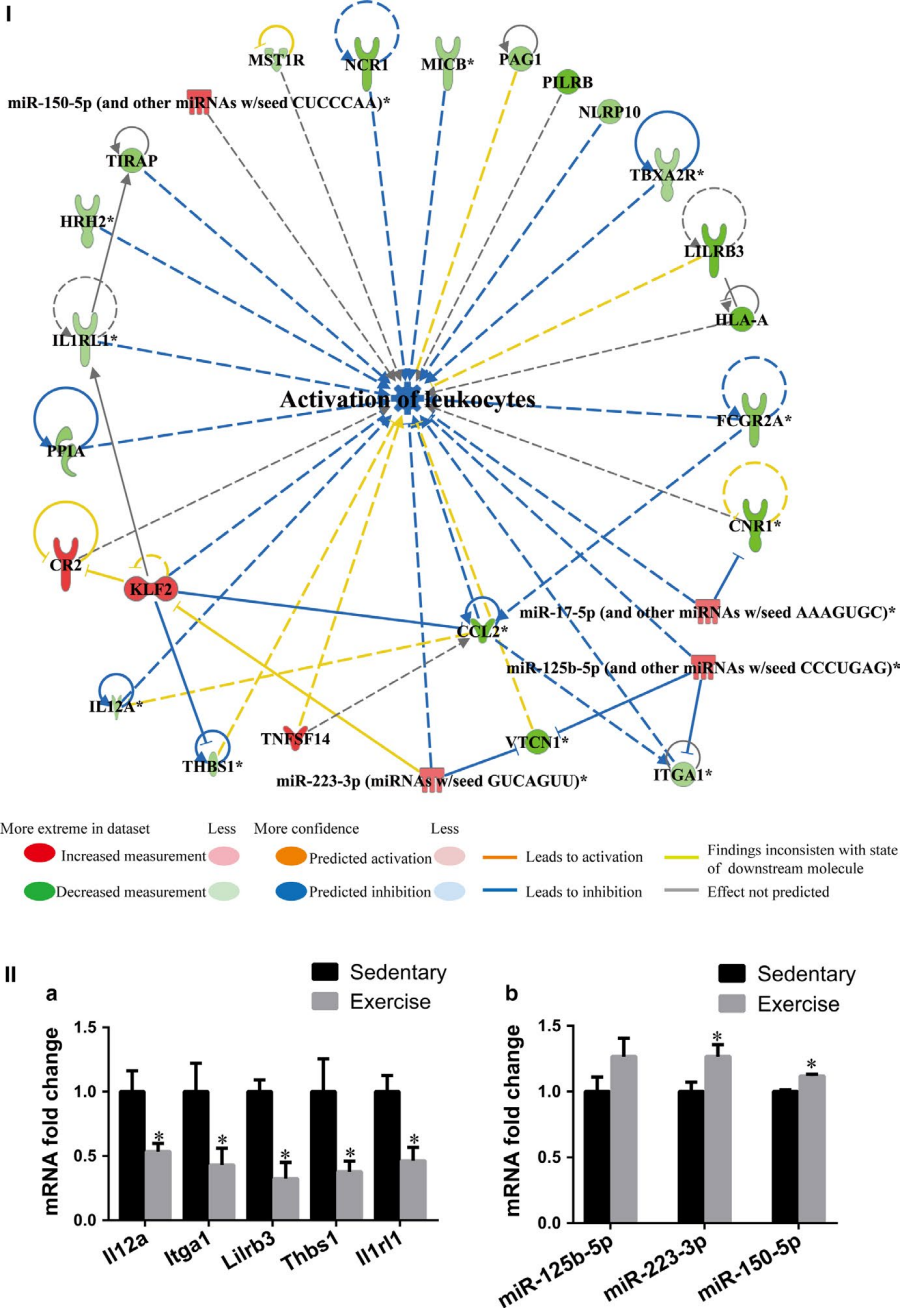
Inhibition of leucocyte activation played a role in the early moderate exercise‐mediated improvement of inflammation. (I) The integrated ingenuity pathway analysis between miRNAs and mRNAs predicted that the activation of leucocytes in the infarct zone was decreased (*P*‐value = 3.51E‐04; *z*‐score = −2.209) in the early moderate exercise MI heart compared with the sedentary MI heart. The regulatory network demonstrates that 27 molecules (20 down‐regulated genes, 3 up‐regulated gene and 4 up‐regulated miRNAs) were included in the regulatory network for the decreased activation of leucocytes. The directive inhibition relationship, which included miR‐125b‐5p for *ITGA1*, *FCGR2A* for *CCL2* and *KLF2* for *CCL2*, was shown to be associated with the inhibition of leucocyte activation. (II) The qPCR quantifications of the expression levels of selected genes included in the decreased activation of the leucocyte network and decreased leucocyte migration network (Figure 7). (a) Selected genes. (b) Selected miRNAs. The animals were trained on the early moderate exercise for two weeks beginning one day after MI. n = 3 per group. *P < .05 vs the sedentary group

**Figure 7:**
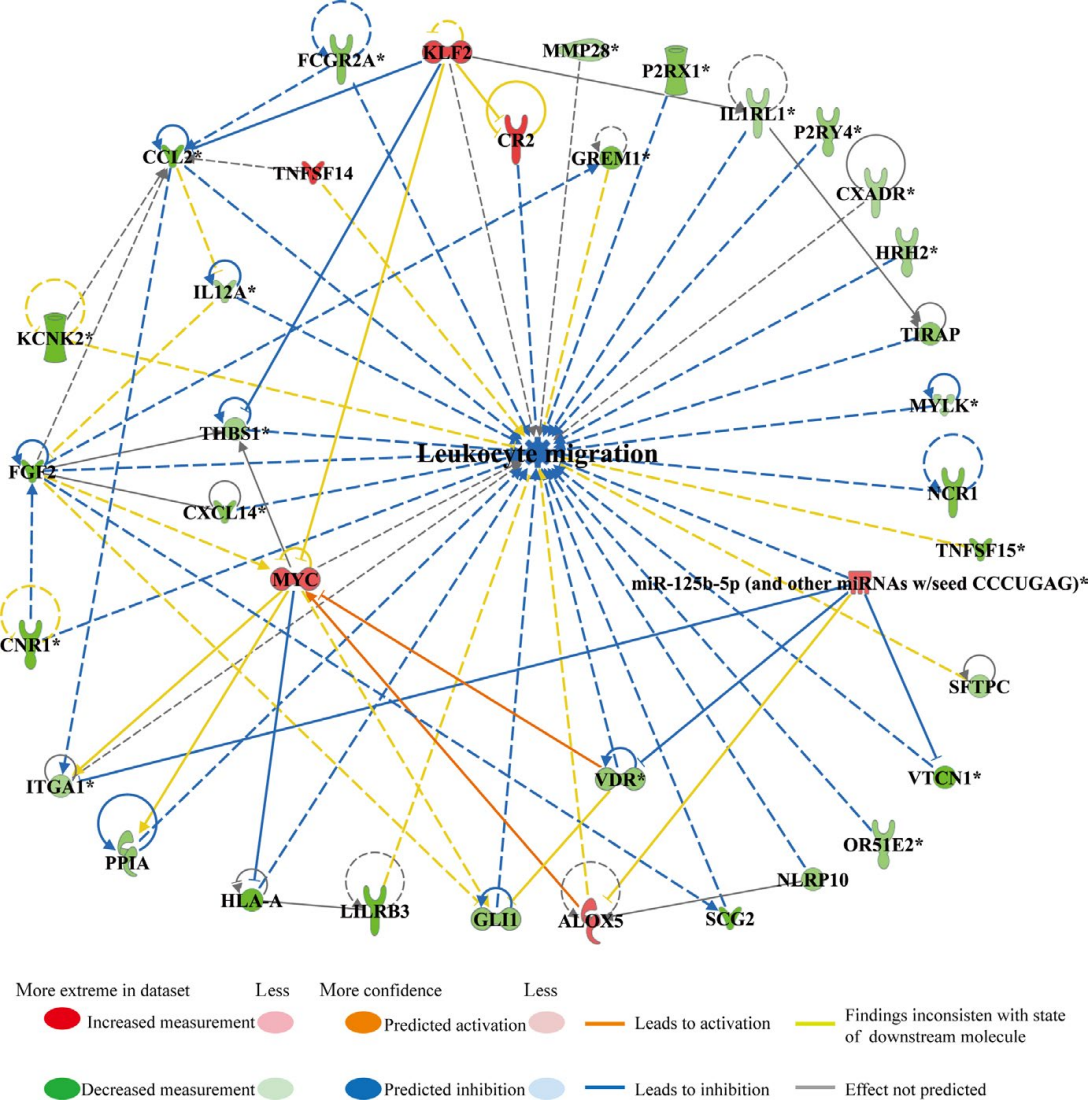
Inhibition of leucocyte migration was included in early moderate exercise‐mediated improvement of inflammation. The integrated ingenuity pathway analysis between miRNAs and mRNAs predicted that leucocyte migration in the infarct zone would be decreased in the early moderate exercise myocardial infarct (MI) heart compared with the sedentary MI heart (*P*‐value = 2.67E‐04; *z*‐score = −2.912). The regulatory network shows that 36 molecules (30 down‐regulated genes, 5 up‐regulated genes and 1 up‐regulated miRNA) were included in the regulatory network for decreased leucocyte migration. The directive inhibition relationship, which included miR‐125b‐5p for *VTCN1* and *VDR* and *KLF2* for *THBS1* and *CCL2*, was revealed to be related to the inhibition of leucocyte migration. The animals were trained on the early moderate exercise for two weeks beginning one day after MI. n = 3 per group

The integrated IPA of miRNA and mRNA expression data also predicted that, compared with that in sedentary MI hearts, the activation of leucocytes, which involved 27 molecules (the down‐regulation of 20 genes and the up‐regulation of 3 genes and 4 miRNAs) was decreased (*P*‐value = 3.51E‐04; *z*‐score = −2.209) in exercise MI hearts, as shown in Appendix S6. In addition, network IPA of the 27 molecules revealed that the down‐regulation of *NCR1*, *MICB*, *PILRB*, *NLRP10*, *TBXA2R*, *LILRB3*, *HLA‐A*, *FCGR2A*, *ITGA1*, *CCL2*, *IL12A*, *PPIA*, *IL1RL1*, *HRH2* and *TIRAP* and the up‐regulation of *KLF2*, miR‐223‐3p, miR‐125b‐5p and miR‐17‐5p led to the inhibition of leucocyte activation. In addition, miR‐125b‐5p was also able to down‐regulate *ITGA1*, and *FCGR2A* was able to down‐regulate *CCL2*, and these both inhibited leucocyte activation. Moreover, *KLF2* was also able to inhibit *CCL2* and led to the inhibition of leucocyte activation (Figure 6I). Indeed, qPCR analysis of the selected representative genes and miRNAs in the infarct zone of early moderate exercise hearts compared with sedentary hearts confirmed that the expression levels of *IL12A*, *ITGA1*, *LILRB3*, *THBS1* and *IL1RL1* were significantly down‐regulated (Figure 6IIa; *P* < .05) and that the expression levels of miR‐223‐3p and miR‐150‐5p were significantly up‐regulated (Figure 6IIb; *P* < .05). Furthermore, leucocyte migration was predicted to have decreased (*P*‐value = 2.67E‐04; *z*‐score = −2.912) in exercise MI hearts compared with sedentary MI hearts, and this involved 36 molecules (30 down‐regulated genes, 5 up‐regulated genes and 1 up‐regulated miRNA), as shown in Appendix S6. Network IPA of the 36 molecules showed that the down‐regulation of *CCL2*, *CNR1*, *CXCL14*, *FCGR2A*, *FGF2*, *GLI1*, *HLA‐A*, *HRH2*, *IL12A*, *IL1RL1*, *MYLK*, *NCR1*, *NLRP10*, *OR51E2*, *P2RX1*, *P2RY4*, *PPIA*, *SCG2*, *THBS1*, *TIRAP*, *VDR* and *VTCN1* and the up‐regulation of *CR2*, *KLF2* and miR‐125b‐5p led to the inhibition of leucocyte migration. In addition, miR‐125b‐5p was able to down‐regulate *VTCN1* and *VDR*, whereas *KLF2* was able to down‐regulate *THBS1* and *CCL2*, and these were revealed to contribute to leucocyte migration inhibition (Figure 7).

## 4 DISCUSSION

Recent research has revealed that post‐MI exercise training is associated with reductions in mortality and reinfarction; therefore, exercise training rather than the traditional instructions to avoid physical activity should be a part of cardiac rehabilitation programmes for MI.^39^, ^40^ However, the timing of the exercise training after MI has not yet been optimized. It has been revealed that moderate exercise beginning 5‐7 days after MI is safe.^8^, ^41^ Much earlier exercise (beginning within 24 hours after MI) has no negative impact on the survival of rats with severe MI.^23^, ^24^ Similar to this observation, our results revealed that an exercise programme that began one day after MI was also safe; the mortality of the exercise group was similar to that of the sedentary group. Our findings strengthen the view that post‐MI moderate exercise training can start very early, even within a day after infarct without causing higher mortality.

Beside the timing, the intensity of the exercise training is also a critical factor for a successful training protocol for MI patients. In our study and studies performed by de Warrd et al^23^ and Bito et al,^24^ the exercise training programme started at same stage, that is, within a day after MI induction. The difference between our study and their studies was the training intensity. While de Warrd, Bito and their colleagues conducted studies using voluntary exercise training (5 km/d on average), the rats in our study ran a moderate 600 m/d. The outcomes of these two exercise intensity grades are different. Studies using a voluntary exercise protocol showed no effect on LV remodelling only showed improvement of global LV dysfunction^23^ and the restoration of cardiomyocyte contraction^24^ in rats that experienced a large MI. In contrast, in our study, the infarct size of the exercise group was significantly smaller than that of the sedentary control group. In parallel, the density of apoptotic cardiomyocytes was also attenuated in the ischaemic myocardium regardless of whether or not they were in the infarct zone, border zone or remote noninfarcted zone. In addition, moderate exercise significantly increased vessel density in both the infarct zone and the border zone in MI compared with that in the sedentary control group. Importantly, the cardiac function of the exercise group was significantly improved compare with that of the sedentary control group. These findings suggest that early moderate exercise can provide more protective effects than early voluntary exercise on the ischaemic myocardium. Similar protective effects were obtained in studies using different training protocols conducted 5‐7 days after MI for 4 weeks.^8^

When MI occurs, LV wall thinning in the infarcted area, the dilation of the ventricular cavity and cardiomyocyte hypertrophy are mortality remodelling phenomena that induce heart failure as the infarct expands.^42^, ^43^ Similar to other reported exercise training protocols,^9^, ^44^ the early moderate exercise protocol that was applied in the present study decreased the collagen content in the infarct zone, increased the thickness of the ventricular wall and reduced cardiomyocyte hypertrophy. Our results suggest that early moderate exercise for two weeks beginning one day after MI provides the same benefits to the MI heart as other reported exercise training protocols in different timeframes via attenuating ventricular remodelling.

Whether cardiac telocytes are involved in exercise‐mediated beneficial effects in MI is an intriguing issue. In the present study, we demonstrated that early moderate exercise significantly increases the number of cardiac telocytes in the border zone. This is not unpredictable, as it has been reported that exercise is able to increase the number of cardiac telocytes in the heart under physiological conditions.^26^ The findings of the present study clearly reveal that an increase in cardiac telocytes in the noninfarcted area is a novel beneficial effect of early moderate exercise on MI. The early moderate exercise‐mediated increase in cardiac telocytes observed in the border zone of MI might have a beneficial effect on the survival of cardiomyocytes in the border zone. The underlying mechanism related to this effect needs to be investigated more deeply in the future. However, our results also documented that early moderate exercise fails to significantly increase the number of cardiac telocytes in the infarct zone. This suggests that the protective effect derived from early moderate exercise is still not enough to protect cardiac telocytes in the infarct zone from cell death. Indeed, our previous study has demonstrated that cardiac telocytes experience serious cell death and are significantly decreased in number in the border zone and especially in the infarct zone during acute MI in rats.^34^, ^35^ They have also suggested that exercise rehabilitation alone is not enough to protect cells in the infarct zone from cell death. A novel strategy for effectively limiting the cell death of cardiomyocytes and cardiac telocytes in the infarct zone is therefore critical for MI regeneration.

Until now, the molecular mechanism underlying the protective effects of MI and the improvement of remodelling attributed to post‐MI exercise training have not been well identified, although many studies in humans have reported changes in effectors in the plasma between exercise and sedentary groups. The IPA results revealed that the TGFB1 regulatory network in the infarct zone of moderate exercise hearts but not of sedentary hearts predicts inhibition. IPA identified a total of 74 genes involved in the regulatory network of TGFB1 inhibition. In this TGFB1 pathway‐related regulatory network, upstream IPA predicted that *SP1*, *SMAD4* and *SMAD3*, which are direct downstream genes regulated by TGFB1, are inhibited, whereas *SMAD7*, the inhibitor of the TGFB1 pathway, is activated. Moreover, qPCR analysis of selected representative genes doubly confirmed that, compared with those in sedentary MI hearts, the expression levels of *SMAD3* and *MAPK14* are down‐regulated and the expression levels of *TGFB1* and *FN1* tend to be down‐regulated in moderate exercise MI hearts. Based on IPA, predicted activation and inhibition relationships identified in the literature and from high‐power confident statistical analyses,^45^ and qPCR analysis of genes that are representative of the changes and the significance of the selected predicted function (*P* < .001 and *Z* > 2), we have confidence that the present results were selected according to these standards. In fact, the up‐regulation of the TGFB1 pathway is known to be related to cardiac fibrosis during MI and cardiac remodelling.^46^, ^47^, ^48^ Therefore, our results suggest that the inhibition of the TGFB1 pathway‐regulated network, which includes 74 genes, as shown in the present study, is an important molecular mechanism for the early moderate exercise‐mediated improvement of cardiac fibrosis and cardiac remodelling in MI. Further interventional studies of genes that are involved in the identified network may allow us to identify novel targets and effective exercise protocols for the inhibition of pathological fibrosis and remodelling after MI.

It has been well established that the inflammatory response is beneficial for MI healing in the early stages of infarct, but it is deleterious in the late phase of scar formation and LV remodelling.^49^ In the present study, the density of pro‐inflammatory cells (CD45^+^ leucocytes and CD68^+^ macrophages) was significantly decreased, and the density of anti‐inflammatory cells (CD206^+^ macrophages and CD163^+^ macrophages) was significantly increased in early moderate exercise MI hearts compared with sedentary MI hearts. Therefore, early moderate exercise for two weeks beginning one day after MI may attenuate the inflammatory response in the infarct zone via decreasing leucocyte and pro‐inflammatory macrophage infiltration and increasing anti‐inflammatory macrophage infiltration. Indeed, the results of our miRNA‐mRNA paired‐match profile integrated IPA revealed that the activation and migration of leucocytes are decreased in the infarct zone of early moderate exercise hearts compared with sedentary hearts. Furthermore, qPCR analysis of the selected representative genes and miRNAs (down‐regulation of *LILRB3*, *ITGA1*, *IL12A*, *IL1RL1* and *THBS1* and up‐regulation of miR‐223‐3p and miR‐150‐5p) was consistent with IPA predictions. A total of 27 genes were identified as activators of leucocytes, and 36 genes were found to be involved in leucocyte migration; down‐regulated *MICB*, *MST1R*, *PAG1* and *TBXA2R* and up‐regulated miR‐150‐5p, miR‐17‐5p and miR‐223‐3p are unique in that they activate leucocytes, whereas down‐regulated *CXADR*, *CXCL14*, *FGF2*, *GLI1*, *GREM1*, *KCNK2*, *MMP28*, *MYLK*, *OR51E2*, *P2RX1*, *P2RY4*, *SCG2*, *SFTPC*, *TNFSF15* and *VDR* and up‐regulated ALOX5 and MYC are unique in that they are involved in leucocyte migration. Further functional study of the identified genes might help us tailor targets to regulate the balance of the suitable infiltration of leucocytes, M1 macrophages and M2 macrophages in the infarct zone to facilitate a protective inflammatory response rather than a pathological inflammatory response. Because the density of M1 macrophages (CD68^+^) in the infract zone of moderate exercise hearts (starting after 1 week post‐MI) was similar to that in sedentary control hearts,^9^ and considering that early moderate exercise for two weeks beginning one day after MI can decrease leucocyte and M1 macrophage infiltration and increase M2 macrophage infiltration in the infarct zone, we propose that early moderate exercise conducted during the early inflammation stage (such as one day after MI) of MI might facilitate improvements in the pathological inflammatory response in the infarct zone via the inhibition of pro‐inflammatory cell infiltration, the accelerated infiltration of M2 macrophages and phenotype switching from M1 to M2 macrophages. Indeed, exercise training inhibits the inflammatory response in adipose tissue via both the suppression of macrophage infiltration and the acceleration of phenotype switching from M1 to M2 macrophages.^50^

Several miRNAs have been shown to control important processes that contribute to the pathophysiological consequences of MI.^51^ In the present study, integrated IPA revealed that the up‐regulation of miR‐17‐5p, miR‐125b‐5p, miR‐223‐3p and miR‐150‐5p plays a role in the decreased activation of leucocytes and that the up‐regulation of miR‐125b‐5p plays a role in decreased leucocyte migration. Indeed, other studies have shown that miR‐17‐5p,^52^ miR‐125b‐5p,^53^ miR‐223‐3p^54^ and miR‐150‐5p^55^ can relieve the inflammatory response of MI via down‐regulating their target genes, namely ASK1, CCL3, IRAK1 and CXCR4, respectively. The findings of the present study further confirm the important roles and uncover a novel miRNA‐mRNA regulatory network of these miRNAs in early moderate exercise training‐mediated anti‐inflammatory effects in MI.

However, the present study only focused on genes that were predicted by IPA to significantly activate and inhibit functions and pathways by IPA, and the present study cannot answer why early moderate exercise leads to the changes in the above genes and miRNAs. However, adaptive responses to energy metabolism might be one of the critical factors; the result of our miRNA‐mRNA paired‐match profile integrated IPA did not predict that the entire expression profiles of energy metabolic function and energy metabolism‐related pathways are activated and inhibited significantly in exercise‐trained MI hearts compared with sedentary hearts. However, the expression of regulatory genes that are critical for the fatty acid metabolism of the primary energy source in healthy adult hearts,^56^, ^57^, ^58^ such as medium‐chain acyl‐CoA dehydrogenase, lipoprotein lipase, peroxisome proliferator‐activated receptor alpha and gamma, and the glucose uptake and utilization regulation gene solute carrier family 2 member 4^56^, ^59^ were found to be increased in exercise MI hearts compared with sedentary hearts (data not shown). This suggests that maintaining fatty acid metabolism as well as glucose uptake and utilization is an important adaptive response mediated by early moderate exercise in MI hearts and might play an important role as an upstream effector to initiate changes in related gene expression, such as that of the genes and miRNAs identified in the present study, as well as others. Therefore, the detailed molecular mechanism of energy metabolism as an upstream target of the adaptive response mediated by early moderate exercise in MI hearts needs to be investigated in the future.

In the present study, MI rats were trained on treadmills, and this is highly similar to aerobic cardiac rehabilitation programmes for humans in which training is predominantly performed in the clinic. Recently, an early mobilization exercise programme beginning 12 hours after MI was shown to improve health‐related quality of life in humans.^22^ Therefore, the early moderate exercise protocol that was applied in the present study might also be applicable for designing a clinical trial protocol for improving cardiac inflammation and ventricular remodelling after MI in humans. However, the difference in many aspects between rodent and human in cardiovascular physiology should be in mind before transposing the protocol to humans. Furthermore, the early moderate exercise training window identified protective effects for MI healing, cardiac remodelling, the maintenance of cardiac telocytes and gene regulatory networks for the inhibition of the TGFB1 pathway, and leucocyte activation and migration; these findings might help us further tailor precise cardiac rehabilitation programmes to humans.

## CONFLICT OF INTEREST

The authors declare that they have no competing interests.

## AUTHOR CONTRIBUTIONS

ZL DL YC YL KZ HC and XZ performed most of the experiments and analysed data; RH ZY HZ QP and XQ contributed to discussion and manuscript writing; and DC conceived and designed this work and wrote the manuscript.

## Data Availability

The data that support the findings of this study are available on request from the corresponding author.
