# Supplementary material for: Early moderate exercise benefits myocardial infarction healing via improvement of inflammation and ventricular remodelling in rats
Source: J Cell Mol Med. 2019 Oct 15;23(12):8328–42. doi: 10.1111/jcmm.14710 (PMC6850916; doi:10.1111/jcmm.14710)
Supplement: Supplementary file 1 [file JCMM-23-8328-s001.docx]

**Materials and Methods**

**Animals**

Three-month-old female Sprague-Dawley rats (250-300 g) were utilized in the present study. The rats were housed for 2 weeks to allow them to adapt before experimentation. They were provided with food and water *ad libitum*. Animal care, surgery and handling procedures were performed according to regulations established by the Ministry of Science and Technology of the People’s Republic of China ([2006] 398) and approved by the Jinan University Animal Care Committee.

**Myocardial infarction studies**

MIs were generated in three-month-old female Sprague-Dawley rats as previously described [1,2]. Briefly, the rats were anesthetized with ketamine (100 mg/kg) and underwent a left intercostal thoracotomy. The left anterior descending coronary artery (LAD) was identified and then ligated directly below the left atrial appendage with 8-0-gauge nylon sutures. The presence of pallor and abnormal movement of the LV confirmed LAD occlusion. The chest wall was then closed, the lungs were inflated, the rat was extubated, and the thoracotomy was closed. After recovery, the rats were returned to the animal facility. At the end of the study, the rats were euthanized, and their hearts were harvested. The tissues were used for RNA isolation, fixed with 4% paraformaldehyde, embedded in paraffin wax and sectioned.

**Treadmill** **exercise**

All animals were habituated to a motorized treadmill by running following a gradual acceleration protocol (0° grade; 10 m/min for 5 min, 15 m/min for 5 min, and 20 m/min for 20 min) each day for 2 weeks. Following 2 days of rest, the rats were randomly divided into two sets: set-1, a sedentary control group (n=20) and a moderate exercise group (n=23); set-2, a sedentary control group (n=7) and a moderate exercise group (n=10). Both groups underwent LAD ligation to induce MI as described above. The rats in the moderate exercise group ran on the treadmill beginning one day post-MI for 2 weeks using the moderate exercise protocol, which was set at 20 m/min for 30 min per day (hereafter called the exercise group) [25]. The rats in the sedentary control group were allowed to be sedentary in their cages for 2 weeks after MI (Fig. 1I). The set-1 animals were used for all observations except the cardiac function analysis, while the set-2 animals were used to analyze of cardiac function. Both sets of animals were included to analyze mortality rate.

**Echocardiography**

Transthoracic echocardiograms were performed and recorded in rats. The experimental rats were anesthetized with ketamine (100 mg/kg, i.p.). The echocardiographic parameters were then collected using an Acuson Sequoia 256c ultrasound system equipped with a 13-MHz linear transducer from a Vevo 770 echocardiogram (VisualSonics, Canada). Briefly, the anterior chest wall was shaved, and the rat was placed in a left lateral decubitus position. A rectal temperature probe was inserted, and the body temperature was carefully maintained between 37°C and 37.5°C on a heating pad throughout the study. Parasternal long-axis, parasternal short-axis and 2 apical four-chamber views were collected in 2D-M-mode. The systolic and diastolic anatomic parameters were obtained from M-mode tracings at the mid-papillary level.

**Masson’s trichrome staining**

Masson’s trichrome staining was conducted as previously described [1,2].Briefly, paraffin sections were dewaxed and rinsed with water using routine protocols. After iron hematoxylin staining (7 min), the sections were rinsed with distilled water, differentiated with ethanol containing 1% hydrochloric acid (30 s), and rinsed with running water (5 min), followed by a final rinse with distilled water. Thereafter, the sections were stained with Ponceau acid fuchsin (5 min) and rinsed with distilled water. After differentiation using a phosphomolybdic acid solution (5 min), the sections were sequentially stained with aniline blue (5 min) and 1% acetic acid (1 min). After staining, the sections were dehydrated, cleared in xylene, and then mounted with resinene. Collagen was stained blue in the sections. Representative sections of the intact heart acquired from the mid-papillary level were stained and photographed. The infarct size was analyzed and presented as the percentage of blue stained for total LV, and the collagen area of the infarct zone (CAIZ) was analyzed as the ratio of the area of blue staining vs. the area of the infarct zone using Image-Pro Analyzer 6.0 software (Media Cybernetics, USA). The wall thickness of the border zone of the left ventricle (WTBZ) and the thickness of the infarct myocardium of the left ventricle (TIM) are schematically illustrated in Fig.2IIIa. Both structures were measured using ImageJ 1.22 software (National Institutes of Health, USA).

**TUNEL assay**

The TUNEL assay (Roche, 11684817910) was applied to detect apoptotic cells. The paraffin sections at the mid-papillary level of the hearts were pretreated with 10μg/ml proteinase K diluted with 10 mM Tris/HCl buffer (pH 7.4) for 15 min. After being rinsed with PBS, the sections were incubated with 50μL of TUNEL reaction mix at 37°C for 60 min, and then the sections were washed with PBS. For the negative controls, the terminal transferase component was omitted from the TUNEL reaction mix. Then, the sections were incubated with 50 μL of converter-peroxidase for 30 min at 37°C. Diaminobenzidine was used as the chromogen. All slides were scanned with the digital image analysis system Pannoramic MIDI II (3DHISTECH, Hungary) at 40× objective.

**Immunohistochemistry staining**

The immunofluorescence assessments were carried out on deparaffinized and rehydrated sections obtained from the mid-papillary level. After deparaffinization and washing, the slides were pretreated with sodium citrate buffer or pronase-mediated antigen retrieval. The sections were then incubated with primary antibodies: rabbit anti-rat CD45 (1:300, Abcam, ab10558), mouse anti-rat CD68 (1:100, AbDSerotec, MCA341R), rabbit anti-rat CD206 (1:200, Abcam, ab64693), and mouse anti-rat CD163 (1:100, AbDSerotec, MCA342R) overnight at 4°C. After washing, the sections were incubated with the appropriate Alexa-Fluor 488 or 555-conjugated secondary antibodies (1:1000) for 1 h and then incubated with 4',6-diamidino-2-phenylindole (DAPI) nuclear counterstain. The images were detected at 40× magnification using a Carl-Zeiss confocal microscope. Only nucleated antibody-positive cells were counted. Data were presented as total positive cells per high power field in the infarct zone.

For the cardiomyocyte cross-sectional area (CSA) analysis, hydrated heart sections were incubated with Alexa Fluor 488-conjugated wheat-germ agglutinin (5 μg/mL Invitrogen, W11261) for 10 min at room temperature. After washing and blocking, the sections were incubated with cardiac troponin T (1:100, Invitrogen, MA5-12960) overnight at 4°C. The donkey anti-mouse Alexa-Fluor 555-conjugated secondary antibodies (1:1000) were applied, and all slides were counterstained with DAPI. For each sample, the CSA was measured in 50 cardiomyocytes in both the border zone and in the remote zone under a double-blinded manner. The CSA was measured only in regions where cardiomyocytes met the following 3 criteria: cellular cross-section is present; visible nuclei located in the center of the cell; and intact cell borders. The images were detected at 40× magnification using a confocal laser microscope and analyzed using ImageJ software.

To detect angiogenesis in the infarcted zone and border zone, tissue sections were immunostained with von Willebrand factor (vWF). Hydrated sections were treated with pronase-mediated antigen retrieval after blocking endogenous peroxidase activity using 3% hydrogen peroxide diluted in PBS. Then, the sections were incubated with rabbit anti-rat vWF (1:300, Sigma, F3520) overnight at 4°C after blocking nonspecific binding with 1% BSA-blocking solution. The sections were washed with PBS three times and then incubated with goat anti-rabbit HRP-conjugated secondary antibodies (1:1000, Invitrogen, 31460) for 1 h. Diaminobenzidine was used as the chromogen. The number of vWF^+^ blood vessels present in the entire infarct zone and border zone were photographed and counted under a microscope in a double-blinded manner. Data were presented as the total capillary density per mm^2^ in the infarcted zone and border zone.

To detect cardiac telocytes (CTs) in the infarcted zone and border zone, CD34 and PDGFRα were used as markers for the semi-quantitative analysis of CTs [4,5]. Briefly, the hydrated sections were pretreated with Tris-EDTA buffer mediated antigen retrieval. Then, the sections were incubated with rabbit anti-rat PDGFRα (1:100, Abcam, ab203491) and goat anti-rat CD34 (1:50; R&D Systems, AF4117) overnight at 4°C after blocking with 1% BSA solution. After washing, the sections were incubated with the donkey anti-rabbit Alexa-Fluor 555 and donkey anti-goat Alexa-Fluor 488-conjugated secondary antibodies (1:1000) for 1 h and then incubated with DAPI nuclear counterstain. In this study, CTs were designated PDGFRα^+^/CD34^+^ cells with a DAPI^+^ nucleus and a very small cell body (piriform/spindle/triangular) with an extremely long and thin protrusion (length ≥60 μm). Images (100×) of the infarct zone were obtained using fluorescence microscopy (Wetzlar GmbH- DM4000B). The cell density in the infarct zone and the standard deviation were used for the semi-quantitative analysis.

**Whole transcriptome sequencing for mRNA**

Total RNA was extracted from the infarcted zone of the moderate exercise group and the sedentary control group using QIAGEN RNeasy Kits (QIAGEN, 217004) following the manufacturer's protocol. RNA integrity was evaluated using an Agilent 2100 Bioanalyzer. Samples with an RNA integrity number equal to or greater than nine were used for the subsequent analysis. Libraries were constructed using TruSeq Stranded mRNA Library Prep Kit (Illumina, RS-122-2101) according to the manufacturer's instructions. The libraries were sequenced on an Illumina sequencing platform (HiSeqTM4000, Illumina) by the Shanghai Biotechnology Co. (Shanghai, China). The 150 bp paired-end reads were generated. The reads from the moderate exercise group and the sedentary control group were mapped to Rat Genome (Rnor_5.0) using TopHat, and gene expression was estimated as fragments per kb per million reads. Differentially expressed genes were identified using DESeq software. And mRNA differential expression that was equal to or greater than a twofold change was considered an expression difference and was selected for an ingenuity pathway analysis (IPA).

**Small RNA sequencing**

Total RNA was extracted from the infarct zone of the moderate exercise group and the sedentary control group using QIAGEN RNeasy Kits (QIAGEN, 217004). An Agilent 2100 Bioanalyzer was applied for quality testing after purification. The libraries were constructed using TruSeq Small RNA Sample Prep Kit (Illumina, RS200-0024) and sequenced on an Illumina sequencing platform (HiSeqTM4000, Illumina). Basic reads were converted into sequence data (called raw data/reads) by base calling. The low-quality reads were filtered, and reads with 5′-primer contaminants and poly A were removed. Reads without 3′-adapter and insert tags and reads shorter than 15 nt and longer than 41 nt from raw data were filtered to obtain clean reads. For the primary analysis, the length distribution of clean sequences in the reference genome was determined. Clean reads were compared with miRNA databases (miRbase 20.0) to annotate the small RNA sequences. Clean reads with less than 10 copies were removed from the list. Only small RNAs whose precursor and mature sequences perfectly matched known rat miRNAs in the miRbase were considered conserved miRNAs, and known miRNA expression patterns in different samples were analyzed. miRNAs in which the differential expression threshold fold change was greater than two were selected for the IPA.

**mRNA-miRNA integrative IPA**

The selected mRNAs and miRNAs with a log_2_ expression ratio greater than 1 or less than -1 were applied to a further mRNA-miRNA integrative analysis to identify the regulatory networks and disease-function analysis using an IPA (http://www.ingenuity.com).The IPA system allows us to identify the putative targets for the input miRNAs by integrating data on the mRNA pair profile; this system then reveals the related regulatory network and physical and disease functions. The confidence of the miRNA target analysis was set to “highly predicted” and “experimentally observed” in the rats. miRecords, Tarbase and TargetScan were selected to predict the miRNA targets in the IPA system. The highly predicted and experimentally observed target genes that were matched to our input selected differential expression miRNA list between the moderate exercise group and the sedentary control group via IPA were further paired with our selected differential expression mRNA list between the moderate exercise group and the sedentary control group based on the expression pairing function of IPA. The analysis results were applied to a further core-analysis in the IPA system. In the present study, only the analyzed data that were to predicted “decrease” or “increase”, which was indicated by a *p* value less than 0.001 and a Z-score larger than 2 or less than -2, were selected as positive predictors.

**Real-time quantitative PCR**

For the miRNA expression analysis, total RNA was reverse-transcribed with the Mir-X™ miRNA First-Strand Synthesis Kit (Clontech, 638313). The quantitative analysis of miRNA expression was performed using TB Green™ Premix Ex Taq™ II (Takara, RR820A) according to the manufacturer’s instructions. The reaction mixture was composed of 12.5μl of 2× Real-time PCR buffer, 1μl of miRNA specific 5’primers, 1 μl of miRNA 3’ primers, 8.5μl of PCR-grade water and 2 μl of template. Amplifications were performed on a CFX96 real-time PCR system (Bio-Rad, USA) using the following PCR conditions: initial denaturation at 95°C for 30 s followed by 40 cycles of amplification at 95°C for 5 s and 60°C for 30 s. Ct values were averaged and normalized to U6. Relative expression was determined using the 2^-ΔΔCt^ comparative threshold method.

For the mRNA expression analysis, the extracted RNA was reverse-transcribed into first-strand cDNAs using the ReverTra Ace q-PCR RT Master Mix with gDNA Remover (Toyobo, FSQ-301,) according to the manufacturer’s instructions, and the gene expression levels were analyzed using SYBR Green-based real-time PCR. The reaction mixture was composed of 10 μl of SYBR Green PCR Master Mix (Bimake, B21202), 1 μl of each primer, 8 μl of PCR-grade water and 1 μl of the cDNA template. The mRNA-specific primer sequences are shown in supplementary Table S1. Amplifications were performed on a CFX96 real-time PCR system (Bio-Rad, USA) under the following conditions: 95°C for 5 min, followed by 40 cycles of denaturation at 95°C for 15 s and primer annealing and extension at 62°C for 30 s. All cDNA samples were amplified in triplicate and normalized to β-actin expression on the same plate. The results of this study were generated from three rats.

**Statistics**

All measured data are presented as the means±standard errors. Two-tailed Student’s *t*-test was used to calculate the statistical significance between two groups. *P* values<0.05 were considered significant.

**References**

1. **Cai D, Xaymardan M, Holm JM*, et al.*** Age-associated impairment in TNF-alpha cardioprotection from myocardial infarction. *Am J Physiol Heart Circ Physiol*. 2003; 285: H463-9.

2. **Cao L, Zhang L, Chen S*, et al.*** BDNF-mediated migration of cardiac microvascular endothelial cells is impaired during ageing. *J Cell Mol Med*. 2012; 16: 3105-15.

3. **Lachance D, Plante E, Bouchard-Thomassin AA*, et al.*** Moderate exercise training improves survival and ventricular remodeling in an animal model of left ventricular volume overload. *Circ Heart Fail*. 2009; 2: 437-45.

4. **Xiao J, Chen P, Qu Y*, et al.*** Telocytes in exercise-induced cardiac growth. *J Cell Mol Med*. 2016; 20: 973-9.

5. **Zhou Q, Wei L, Zhong C*, et al.*** Cardiac telocytes are double positive for CD34/PDGFR-alpha. *J Cell Mol Med*. 2015; 19: 2036-42.
