# Supplementary material for: Early moderate exercise benefits myocardial infarction healing via improvement of inflammation and ventricular remodelling in rats
Source: J Cell Mol Med. 2019 Oct 15;23(12):8328–42. doi: 10.1111/jcmm.14710 (PMC6850916; doi:10.1111/jcmm.14710)
Supplement: Supplementary file 2 [file JCMM-23-8328-s002.docx]

Supporting Information S2: primer list

| miRNA or gene | Forward primer sequence (5’-3’) | Reverse primer sequence (5’-3’) |
| --- | --- | --- |
| rno-miR-125b-5p | CCTGAGACCCTAACTTGTGA |  |
| rno-miR-223-3p | TGTCAGTTTGTCAAATACCCC |  |
| rno-miR-150-5p | CCCAACCCTTGTACCAGTG |  |
| Tgfb1 | CCTGAGTGGCTGTCTTTTGA | CGTGGAGTACATTATCTTTGCTG |
| Smad3 | CATCCCCGAGAACACTAACTTC | CATCTTCACTCAGGTAGCCAG |
| Smad7 | GTGTTGCTGTGAATCTTACGG | TCGGGTATCTGGAGTAAGGAG |
| Mapk14 | TTACCGATGACCACGTTCAG | CCAGCCCAAAATCCAGAATC |
| Fn1 | TGCCTTCAACTTCTCCTGTG | GACACTAACCACATACTCCACG |
| Il12a | CCAAGTGTCTTAACCAGTCCC | TTGTCCCGTGTGATGTCTTC |
| Itga1 | CAGGTCGGGATTGTACAGTATG | CTGTGTCTATTCCAAGGGCTG |
| Lilrb3 | CAAAGTTTCAGACTCTGGCATCAG | TGCAGGTGTCCAACAATGCT |
| Thbs1 | AGGTGTCGAACACGCTATGG | GTTGCACTTGGCGTTCTTGT |
| Il1rl1 | GACTCACCGTTACCTTCCTG | TTTTCTGCCCTCTGTCACTATG |
