# Supplementary material for: Early moderate exercise benefits myocardial infarction healing via improvement of inflammation and ventricular remodelling in rats
Source: J Cell Mol Med. 2019 Oct 15;23(12):8328–42. doi: 10.1111/jcmm.14710 (PMC6850916; doi:10.1111/jcmm.14710)
Supplement: Supplementary file 3 [file JCMM-23-8328-s003.docx]

**Supporting Information S3: IPA revealed Th2 pathway trends activation and leukocyte extravasation signaling trends inhibition.**

| Canonical pathways | *P*-value | z-score | Associated genes (log2 ratio) |
| --- | --- | --- | --- |
| Th2 pathway | 1.45E-02 | 1.342 | *CD28* (1.967), *FGF2* (-1.527), *HLA-A* (-6.947), *HLA-DQA1* (2.678), *HLA-DQB1* (2.681), *HLA-DB5* (-1.988), *IL12A* (-1.203), *IL1RL1* (-1.062), *TLR9* (1.071) |
| Leukocyte extravasation signaling | 2.46E-02 | -1.134 | *ACTC2* (-1.292), *ACTG2* (-1.464), *CLDN1* (-1.036), *CLDN15* (1.075), *CLDN22* (1.802), *CLDN23* (1.525), *FGFR2* (-1.527), *ITGA1* (-1.076), *MMP28* (-1.031), *MMP8* (2.082), *NOX1* (1.244), *TLR9* (1.071) |

Note: The result is from the IPA canonical pathways analysis of differential mRNAs in infarct zone of MI between the moderate exercise heart and the sedentary heart.
