# Supplementary material for: Early moderate exercise benefits myocardial infarction healing via improvement of inflammation and ventricular remodelling in rats
Source: J Cell Mol Med. 2019 Oct 15;23(12):8328–42. doi: 10.1111/jcmm.14710 (PMC6850916; doi:10.1111/jcmm.14710)
Supplement: Supplementary file 4 [file JCMM-23-8328-s004.docx]

**Supporting Information S4: IPA reveals cardiotoxicity related miRNAs**

| Diseases annotation | *P*- value | Associated miRNAs (log2 ratio) |
| --- | --- | --- |
| Fibrosis of heart | 1.89E-03 | miR-133a-3p (1.348), miR-150-5p (1.288), miR-351-5p (1.174), miR-30b-5p (1.024) |
| Hypertrophy of cardiomyocytes | 5.38E-03 | miR-1-3p (1.962), miR-133a-3p (1.348), miR-150-5p (1.288) |
| Hypertrophy of left ventricle | 2.23E-01 | miR-133a-3p (1.348) |
| Viral myocarditis | 9.13E-03 | miR-499-5p (1.283) |
| Myocardial infarction | 3.10E-02 | miR-1-3p (1.962), miR-133a-3p (1.348), miR-499-5p (1.283) |
| Acute myocardial infarction | 3.63E-02 | miR-1-3p (1.962), miR-133a-3p (1.348) |
| Dilated cardiomyopathy | 3.66E-02 | miR-1-3p (1.962), miR-20a-5p (1.080), miR-30b-5p (1.024) |

Note: The result is from the IPA of differential miRNAs in infarct zone of MI between the moderate exercise heart and the sedentary heart.
