# Supplementary material for: Early moderate exercise benefits myocardial infarction healing via improvement of inflammation and ventricular remodelling in rats
Source: J Cell Mol Med. 2019 Oct 15;23(12):8328–42. doi: 10.1111/jcmm.14710 (PMC6850916; doi:10.1111/jcmm.14710)
Supplement: Supplementary file 5 [file JCMM-23-8328-s005.docx]

**Supporting Information S5: IPA reveal related genes included in inhibition of TGFB1 regulatory network**

| Predicted genes | Predicted state | Downstream gene (log2 ratio) | |
| --- | --- | --- | --- |
|  |  | Increased measurement | Decreased measurement |
| *FN1* | inhibition | *KRT7* (1.760)*, MYC* (1.254) | *ACTA2* (-1.292)*, CCL2* (-2.788)*, FGF2* (-2.426)*, ITGA1* (-1.076)*, TAGLN* (-1.012)*, THBS1* (-1.124) |
| *MAPK14* | inhibition | *FOSL2* (1.072) | *ACTA2* (-1.292)*, CCL2* (-2.788)*, EREG* (-1.551)*, GREM1* (-1.856)*, IL12A* (-1.203)*, MYH11* (-1.560)*, TAGLN* (-1.012)*, VDR* (-1.361) |
| *SP1* | inhibition | *ALOX5* (1.217)*, CR2* (2.604)*, MYC* (1.254)*, TNFSF14* (2.256) | *ACTA2* (-1.292)*, ALOX12* (-1.261)*, BMP7* (-1.525)*, CCL2* (-2.788)*, COL2A1* (-1.572)*, EREG* (-1.551)*, FGF2* (-2.406)*, IL12A* (-1.203)*, MYCN* (-1.059)*, MYH11* (-1.560)*, MYLK* (-1.060)*, TAGLN* (-1.012)*, TBXA2R* (-1.065)*, VDR* (-1.361) |
| *SP3* | inhibition | *TNFSF14* (2.256) | *ACTA2* (-1.292)*, ALOX12* (-1.261)*, COL2A1* (-1.572)*, EREG* (-1.551)*, MYCN* (-1.059)*, MYH11* (-1.560)*, MYLK* (-1.060)*, TAGLN* (-1.012) |
| *ESR1* | inhibition | *ALOX5* (1.217)*, FOSL2* (1.072), *KRT7* (1.760), *MYC* (1.254)*, SOSTDC1* (1.566)*, TCIM* (2.630) | *CA12* (-1.011)*, CCL2* (-2.788)*, CLDN1* (-1.306)*, CNN1* (1.529)*, CXADR* (-1.002)*, DISC1* (-1.187)*, EREG* (-1.551)*, FGFR2* (-1.527)*, FMN1* (-1.233)*, GREM1* (-1.856)*, IL12A* (-1.203)*, KCNMA1* (-2.156)*, MAP2* (-3.078)*, MST1R* (-1.074)*, PCP4* (-2.557)*, PTPRH* (-1.788)*, SLC9A5* (-1.299)*, SOST* (-2.319)*, TDO2*(-1.384)*, TIRAP* (-1.444) |
| *SMAD4* | inhibition | *MYC* (-1.254)*, RGCC* (1.066) | *BMP7* (1.525)*, CCL2* (-2.788)*, EREG* (-1.551)*, GLI1* (-1.391)*, HRH2* (-1.142)*, MST1R* (-1.074)*, MYCN* (-1.059)*, THBS1* (-1.124) |
| *EGR1* | inhibition | *MYC* (1.254) | *ACTA2* (-1.292)*, CCL2* (-2.788)*, COL2A1* (-1.572)*, EREG* (-1.551)*, FGF2* (-2.406)*, GLI1* (-1.391)*, SLC12A5* (-1.525)*, THBS1* (-1.124) |
| *CREBBP* | inhibition | *FOSL2* (1.072)*, KLF2 (1.397), MYC* (1.254) | *BMP7* (1.525)*, COL2A1* (-1.572)*, FGF2* (-2.406)*, GLI1* (-1.391)*, LIG4* (-1.250)*, NTRK3* (-1.552)*, SMOC1* (-1.487)*, TAGLN* (-1.012)*, THRSP* (-1.297)*, WNT5B* (-1.062) |
| *SMAD3* | inhibition | *MYC* (1.254) | *ACTA2* (-1.292)*, CCL2* (-2.788)*, COL2A1* (-1.572)*, EREG* (-1.551)*, FZD8* (-1.078)*, GLI1*(-1.391)*, TAGLN* (-1.012)*, THBS1* (-1.124) |
| *HDAC2* | activation | *ALOX5* (1.217)*, MYC* (1.254)*,* | *COL11A1* (-1.061)*, COL2A1* (-1.572)*, GLI1* (-1.391)*, MYCN* (-1.059)*, SLC12A5* (-1.059)*, TAGLN* (-1.012) |
| *TP73* | activation |  | *ACTA2* (-1.292)*, BMP7* (1.525)*, CCL2* (-2.788)*, DBP* (-1.187)*, EREG* (-1.551)*, FGF2* (-2.406)*, FGFR2* (-1.527)*, MMP28* (-1.013)*, MYCN* (-1.059)*, SCRG1* (-1.138)*, THBS1* (-1.124)*, TNNI1* (-1.109)*, VDR* (-1.361) |
| *SMAD7* | activation | *MYC* (1.254) | *ACTA2 (-1.292), BMP7* (1.525)*, CCL2* (-2.788)*, COL2A1* (-1.572)*, SFTPC* (-1.078)*, TAGLN* (-1.012) |

Note: The results from the IPA upstream analysis for miRNA-mRNA integrating analysis in infarct zone of MI between the moderate exercise heart and the sedentary heart.
