# Supplementary material for: Early moderate exercise benefits myocardial infarction healing via improvement of inflammation and ventricular remodelling in rats
Source: J Cell Mol Med. 2019 Oct 15;23(12):8328–42. doi: 10.1111/jcmm.14710 (PMC6850916; doi:10.1111/jcmm.14710)
Supplement: Supplementary file 6 [file JCMM-23-8328-s006.docx]

**Supporting Information S6: IPA reveals the underlying mechanism for moderate exercise-mediated improvement of the inflammatory response**

| Diseases or Functions Annotation | *P*-value | Predicted Activation State | z-score | Molecules (log2 ratio) |
| --- | --- | --- | --- | --- |
| Activation of leukocytes | 3.51E-04 | Decreased | -2.209 | CCL2(-2.788), CNR1(-2.155), CR2(2.604), FCGR2A(-1.618), HLA-A(-6.947), HRH2(-1.142), IL12A(-1.203), IL1RL1(-1.062), ITGA1(-1.076), KLF2(1.397), LILRB3(-2.366), MICB(-1.228), miR-125b-5p(1.174), miR-150-5p(1.288), miR-17-5p(1.080), miR-223-3p(1.100), MST1R(-1.074), NCR1(-1.820), NLRP10(-1.288), PAG1(-1.291), PILRB(-4.985), PPIA(-1.426), TBXA2R(-1.065), THBS1(-1.124), TIRAP(-1.444), TNFSF14(2.256), VTCN1(-2.262) |
| Leukocyte migration | 2.67E-04 | Decreased | -2.912 | ALOX5(1.217), CCL2(-2.788), CNR1(-2.155), CR2(2.604), CXADR(-1.002), CXCL14(-1.658), FCGR2A(-1.618), FGF2(-2.426), GLI1(-1.391), GREM1(-1.856), HLA-A(-6.947), HRH2(-1.142), IL12A(-1.203), IL1RL1(-1.062), ITGA1(-1.076), KCNK2(-2.333), KLF2(1.397), LILRB3(-2.366), miR-125b-5p(1.174), MMP28(-1.013), MYC(1.254), MYLK(-1.060), NCR1(-1.820), NLRP10(-1.288), OR51E2(-1.331), P2RX1(-1.664), P2RY4(-1.319), PPIA(-1.426), SCG2(-2.830), SFTPC(-1.078), THBS1(-1.124), TIRAP(-1.444), TNFSF14(2.256), TNFSF15(-1.841), VDR(-1.361), VTCN1(-2.262) |

Note: The results from the IPA for miRNA-mRNA integrating analysis in the infarct zone of MI. Z score<-2 indicates a decrease in prediction activation state.
